# Colorectal Carcinoma—An Anomalous Trigger of Adult Hemophagocytic Lymphohistiocytosis

**DOI:** 10.1177/23247096241273131

**Published:** 2024-09-30

**Authors:** Ameish Govindarajan, Frederick Venter, Akriti Chaudhry, Harsimranjit Kaur, Everardo Cobos, Greti Petersen

**Affiliations:** 1 City of Hope Comprehensive Cancer Center, Duarte, CA, USA; 2 Kern Medical, Bakersfield, USA; 3 University of California, San Francisco, Fresno, USA

**Keywords:** colorectal carcinoma, hemophagocytic lymphohistiocytosis, HLH

## Abstract

Hemophagocytic lymphohistiocytosis (HLH) is a rare but often fatal condition characterized by a hyperinflammatory immune response leading to multiorgan failure. It is predominantly observed in the pediatric population and can be classified as familial or acquired HLH. The latter is more common in adults, often associated with malignancy, infection, or autoimmune diseases. Among acquired HLH cases, hematologic neoplasms account for the majority, with only a few isolated reports documenting solid neoplasms as the cause. Herein, we present a case of adult HLH associated with colorectal adenocarcinoma, which, to the best of our knowledge, is only the second reported case of HLH associated with this type of cancer.

## Introduction

Hemophagocytic lymphohistiocytosis (HLH) is a rare but often fatal condition characterized by a hyperinflammatory immune response leading to multiorgan failure. It is predominantly observed in the pediatric population and can be classified as familial or acquired HLH. The latter is more common in adults, often associated with malignancy, infection, or autoimmune diseases. Among acquired HLH cases, hematologic neoplasms account for the majority, with only a few isolated reports documenting solid neoplasms as the cause. Herein, we present a case of adult HLH associated with colorectal adenocarcinoma, which to the best of our knowledge, is only the second reported case of HLH associated with this type of cancer.^1,2^

Although HLH is an under-studied topic, available studies disproportionately focus on the pediatric population, while adult HLH cases are likely underreported due to diagnostic challenges.^3^ Recent years have seen an increase in the incidence of adult HLH.^4^ HLH can be classified as familial or acquired based on the genetic and environmental factors inciting the immune response. Familial HLH is a heritable condition arising from genetic mutations, while acquired HLH has a diverse range of causative factors, with malignancy, infection, and autoimmune disorders being the most common.^5^ Malignancy is responsible for nearly half of all adult HLH cases and has the worst prognosis among all studied forms.^6,7^

Early diagnosis and appropriate management of HLH, regardless of its etiology, are crucial for reducing morbidity and mortality.^8^ Our case report describes a rare case of a 56-year-old male with HLH associated with colorectal carcinoma, highlighting the importance of prompt diagnosis and management.

## Case Presentation

A 56-year-old previously healthy Caucasian male presented to the emergency department with complaints of subjective fevers, chills, non-productive cough, and worsening dyspnea over a period of 1.5 months, accompanied by right-sided abdominal pain. In addition, he reported unintentional weight loss of 20 pounds and a 1.5-week history of melena. The patient had a colonoscopy 4 years ago, revealing a single polyp, but he did not follow-up with a repeat colonoscopy 1 year later. He had no family history of colon cancer.

On physical examination, the patient was found to be tachycardic and tachypneic, with icteric sclera, diffuse anasarca, and a mildly distended abdomen that was tender to palpation. The patient maintained low-grade fevers throughout his hospital stay. Initial laboratory studies revealed a white blood cell count of 22.9, alanine aminotransferase (ALT): 143, aspartate aminotransferase (AST): 575, alkaline phosphatase: 524, lactate dehydrogenase (LDH): 3692, total bilirubin of 6.3, elevated C-reactive protein (CRP) of 22.10, procalcitonin of 3.56, ferritin of 18 605, and triglyceride of 326. Coagulation studies were abnormal, with an elevated prothrombin time (PT) of 21.7 and an elevated D-dimer >5000. (see Table 1)

**Table 1.** Significant Laboratory Findings

Significant laboratory findings	
White blood cell (4-11 × 10^3^/mcL)	22.9
Red blood count (4.26-5.80 × 10^6^/mcL)	3.93
Hemoglobin (13.2-17.4 g/dL)	12.8
Platelets (150-450 × 10^3^/mcL)	60
Aspartate transaminase (15-37 unit/L)	575
Alanine transaminase (13-61 unit/L)	143
Alkaline phosphatase (45-117 unit/L)	524
Serum triglyceride (<150 mg/dL)	326
Total bilirubin (0-1 mg/dL)	6.3
D-dimer (<500 ng/mL)	5000
Prothrombin time (12.5-14.3 seconds)	21.7
Partial thromboplastin time	36.4
(25.4-37.6 seconds)	
Fibrinogen (150-450 mg/dL)	144
Lactate dehydrogenase (87-241 unit/L)	3692
Ferritin (20-300 ng/mL)	18 605
C-reactive protein (<0.30 mg/dL)	22.10
Lactic acid plasma (0.4-2.0 mmol/L)	7.5
Procalcitonin (<0.50 ng/mL)	3.56
Sodium (136-145 mmol/L)	133
Potassium (3.5-5.1 mmol/L)	5.5
Chloride (98-107 mmol/L)	92
CO_2_ (21-32 mmol/L)	22
Calcium (8.5-19.1 mg/dL)	7.2
BUN (7-18 mg/dL)	96
Creatinine (0.67-1.17 mg/dL)	3.03
EGFR (>60 mL/min/1.73 m^2^)	22
IL-2 receptor (532-1891 pg/mL)	15 282
CEA (<2.5 ng/mL)	1633.2
CA 19-9 (<34 units/mL)	527 967

In this case, although the patient did not have significant anemia (hemoglobin of 12.8), the high ferritin levels prompted consideration of HLH. During hospitalization, the patient’s presentation with a quick Sequential Organ Failure Assessment (qSOFA) score of 3 necessitated immediate treatment for suspected sepsis, including empiric broad-spectrum antibiotics. The qSOFA score, used to identify patients at risk of sepsis, includes criteria, such as altered mental status, respiratory rate ≥ 22 breaths/min, and systolic blood pressure ≤ 100 mmHg. This approach was justified given the high-risk indicators, such as altered mental status, elevated respiratory rate, and low systolic blood pressure.

Initial imaging included an abdominal ultrasound that demonstrated a large nodular liver with innumerable hypodense masses. Abdominal computed tomography (CT) with and without contrast confirmed severe hepatomegaly and hepatic steatosis in a background of cirrhosis along with innumerable lesions throughout the liver. Also noted were multiple pathologically enlarged lymph nodes, including portal hepatic, epigastric, paraesophageal lymph nodes suggesting widespread metastasis, prompting further evaluation of the underlying malignancy.

Ultrasound-guided biopsy of one of the liver lesions suggested metastatic adenocarcinoma with malignant cells staining positive for CDX2, CK7, and weakly positive for CK20, suggesting a gastrointestinal origin of malignancy. Tumor marker levels were elevated as expected in the setting of colorectal carcinoma, with a carcinoembryonic antigen (CEA) level of 1633 and cancer antigen 19-9 (CA 19-9) of 527 967.

The patient’s clinical status began to deteriorate throughout his hospital stay, even with intravenous antibiotics initiated due to concerns of sepsis. Multiorgan failure ensued, as evidenced by laboratory changes indicating a decline in kidney function, hepatic failure, and respiratory failure. The patient was transferred to the intensive care unit due to increasing oxygen demands. With significantly elevated ferritin levels and other symptoms, HLH was suspected, and interleukin-2 receptor (IL-2r) (CD25s) was sent to confirm the diagnosis. Unfortunately, the patient’s poor performance status precluded HLH-specific therapy, and he opted for comfort care. He was discharged to a hospice facility the next day. One week after discharge, the IL-2r levels were reported at 15 282, confirming the diagnosis of HLH.

Despite early consideration of HLH based on the elevated ferritin levels, the patient’s rapid clinical deterioration and multiorgan failure precluded the initiation of targeted HLH therapy. This highlights the challenge in managing critically ill patients where the diagnosis of HLH, although timely, may not alter the immediate clinical course due to the patient’s acute condition.

## Discussion

Hemophagocytic lymphohistiocytosis is a rare and life-threatening disorder characterized by a dysregulated immune response that leads to multiorgan failure.^7^ While much of the research on HLH has focused on the hereditary form of the disease, the acquired form is now becoming increasingly recognized.^7^ The pathogenesis of acquired HLH is complex and not yet fully understood, but it is thought to involve multiple factors.^7^ One proposed mechanism for malignancy-associated HLH (M-HLH) is that the tumor cells cause extreme inflammation and persistent antigen stimulation, thus disrupting immune homeostasis.^8^ Unchecked cytotoxic T cells and macrophage activation lead to excessive release of cytokines, inducing inflammation. This inflammation, along with the proliferation and infiltration of these cells into organs, eventually causes organ failure.^9,10^

Diagnosing HLH can be challenging due to its nonspecific clinical and laboratory findings, which can mimic other diseases, such as sepsis or autoimmune disorders.^10,11^

In this case, although the patient’s hemoglobin was 12.8 g/dL, the elevated ferritin levels raised suspicion for HLH. The patient’s initial qSOFA score of 3 warranted immediate empiric treatment for suspected sepsis due to altered mental status, high respiratory rate, and low blood pressure.

Common clinical manifestations include fever, malaise, hepatosplenomegaly, cytopenia, hyperferritinemia, hypertriglyceridemia, and elevated LDH.^7,12^ However, the hallmark feature of HLH is its rapid progression and clinical deterioration without appropriate treatment.^7^ In 1991, the Histiocyte Society proposed diagnostic criteria for HLH, which have since been updated to the HLH 2004 diagnostic criteria, requiring 5 of the 8 criteria to be fulfilled to diagnose HLH.^12^

Two of the 8 criteria, ferritin and IL-2r, serve as markers of generalized inflammation.^13^ Interleukin-2 receptor is a heterotrimer transmembrane protein that is upregulated on activated T cells and has an important diagnostic value for HLH; IL-2r > 2400 U/mL has 100% sensitivity, and IL-2r > 10 000 U/mL confers 93% specificity, with higher levels observed in M-HLH.^14^ However, the turnaround time for IL-2r testing is often long and may delay diagnosis. Conversely, ferritin is a readily available laboratory indicator of inflammation. While previously a ferritin level above 10 000 was considered highly specific for HLH,^15^ recent studies have shown extreme elevations in ferritin may also occur in other disease processes, such as liver disease and hematologic malignancy, thereby decreasing the specificity of ferritin as it pertains to HLH.^16^ Nonetheless, ferritin remains a valuable prognostic factor, with a level exceeding 50 000 correlating with a 30-day mortality.^15^

It should be noted that the HLH 2004 criteria were established based on pediatric studies, and separate guidelines have not been proposed for adults with acquired HLH. These criteria have been criticized for being too stringent and potentially excluding some adult HLH patients. In a retrospective study, Tamamyan et al^8^ constructed an extended 18-variable diagnostic criteria to include clinical and laboratory features identified in M-HLH patients, which resulted in identifying more individuals that were not originally given HLH-specific treatment because they failed to meet HLH 2004 requirements.

Malignancy-associated HLH is a particularly lethal form of HLH and yet research in this field is scarce.^17^ It has been reported that up to 1% of patients with underlying malignancy may develop HLH.^5,8,10^ This can occur at the time of diagnosis, during chemotherapy or even after the malignancy is controlled, with those having active malignancy at the highest risk.^5,8,10^ In a systematic review, 93.7% of the malignancies associated with HLH were hematologic neoplasms, while only 3.1% were discovered in solid malignancies, such as prostate, breast, lung, and hepatocellular carcinoma.^5^ To the best of our knowledge, only one case of HLH associated with colorectal carcinoma has been reported, and it involved a different disease course than the one presented here.^18^

Without treatment, M-HLH is known to have an aggressive course, with a median overall survival of 1.13 months.^15^ However, the treatment guidelines for adults have been extrapolated from the pediatric guidelines, just like the diagnostic criteria. The initial approach to treatment for HLH patients should be supportive care to stabilize the patient.^5^ Thereafter, corticosteroids with or without intravenous immunoglobulin (IVIG) are typically included in initial treatment regimens, with the aim to suppress the exaggerated immune response before starting cancer-specific treatment.^5^ Unfortunately, many M-HLH patients do not receive HLH-directed therapy due to delay in diagnosis, often resulting in multiorgan dysfunction, as seen in our patient. The irreversible damage renders the necessary lymphocytic or cytotoxic therapy ineffective.^6^

## Conclusion

Hemophagocytic lymphohistiocytosis is a serious medical condition characterized by an overactive immune system. Due to the severity of this disease, an early and accurate diagnosis is crucial for optimal patient outcomes. Unfortunately, due to the non-specific nature of the symptoms and delays in testing, many patients are at risk of developing multiorgan failure. While most cases of acquired HLH secondary to malignancy are associated with hematologic cancer, it is important to maintain a high degree of suspicion for HLH in patients with solid tumors when clinically appropriate.
